# Mid-term follow-up surgical results in 284 cases of clival chordomas: the risk factors for outcome and tumor recurrence

**DOI:** 10.1007/s10143-021-01576-4

**Published:** 2021-10-08

**Authors:** Jiwei Bai, Mingxuan Li, Jianxin Shi, Liwei Jing, Yixuan Zhai, Shuheng Zhang, Junmei Wang, Peng Zhao, Chuzhong Li, Songbai Gui, Yazhuo Zhang

**Affiliations:** 1 Beijing Neurosurgical Institute, Capital Medical University, Beijing, China; 2 Department of Neurosurgery, Beijing Tiantan Hospital, Capital Medical University, Beijing, China; 3 China National Clinical Research Center for Neurological Diseases, Beijing, China; 4 Division of Cancer Epidemiology and Genetics, National Cancer Institute, NIH, DHHS, Bethesda, MD USA; 5 Department of Health Statistics, Shanxi Medical University, Taiyuan, China; 6 Department of Neurosurgery, First Affiliated Hospital, Zhengzhou University, Zhengzhou, China; 7 Department of Neurosurgery, Anshan Central Hospital, Anshan, China

**Keywords:** Chordoma, Prognosis, Radiation therapy, Skull base, Surgical approaches

## Abstract

**Objective:**

Skull base chordoma (SBC) is rare and one of the most challenging diseases to treat. We aimed to assess the optimal timing of adjuvant radiation therapy (RT) and to evaluate the factors that influence resection and long-term outcomes.

**Methods:**

In total, 284 patients with 382 surgeries were enrolled in this retrospective study. Postsurgically, 64 patients underwent RT before recurrence (pre-recurrence RT), and 47 patients underwent RT after recurrence. During the first attempt to achieve gross-total resection (GTR), when the entire tumor was resected, 268 patients were treated with an endoscopic midline approach, and 16 patients were treated with microscopic lateral approaches. Factors associated with the success of GTR were identified using χ^2^ and logistic regression analyses. Risk factors associated with chordoma-specific survival (CSS) and progression-free survival (PFS) were evaluated with the Cox proportional hazards model.

**Results:**

In total, 74.6% of tumors were marginally resected [GTR (40.1%), near-total resection (34.5%)]. History of surgery, large tumor volumes, and tumor locations in the lower clivus were associated with a lower GTR rate. The mean follow-up period was 43.9 months. At the last follow-up, 181 (63.7%) patients were alive. RT history, histologic subtype (dedifferentiated and sarcomatoid), non-GTR, no postsurgical RT, and the presence of metastasis were associated with poorer CSS. Patients with pre-recurrence RT had the longest PFS and CSS, while patients without postsurgical RT had the worst outcome.

**Conclusion:**

GTR is the goal of initial surgical treatment. Pre-recurrence RT would improve outcome regardless of GTR.

**Supplementary Information:**

The online version contains supplementary material available at 10.1007/s10143-021-01576-4.

## Introduction

Chordoma is a rare bone malignancy with an incidence rate of 0.08 and 0.04 per 100,000 in the USA and Europe [11, 20] and in Taiwan [13], respectively. Skull base chordomas (SBCs) account for 32% of all chordomas [20]. Local recurrence is common with a late recurrence rate > 50% [8, 10, 14, 25, 30]. The average survival after surgery with or without radiation therapy (RT) is approximately 7.7 years [35]. Because of the deep location and close proximity to vital structures, surgical treatment is a challenge for SBCs with the total resection rate ranging from 0 to 73.7% [8]. The endoscopic midline approach (EMA) for SBCs yields better or similar resections than the microscopic lateral open approach (MLOA) [12, 26]. Factors that influence resection with EMA still need to be clarified.

Although RT is recommended as an adjuvant treatment to surgery [24], the use of RT remains controversial [7, 8, 23, 40]. In clinical practice, it is not clear whether and when RT should be administered [23, 31], especially when the tumor was gross-total resected [33]. Hence, a comprehensive investigation of the optimum management protocol conducted in a large study is warranted [8, 14].

Our group, the first neuroendoscopic group in China, was established in Beijing Tiantan Hospital in 1998 and has since then focused on skull base diseases [18]. We have used both EMA and MLOA to resect SBCs during the past two decades. In this study, we retrospectively analyzed the clinical data of 284 patients with SBCs who were treated by our single group to identify factors for achieving complete resection and the optimal timing for RT.

## Methods

### Patients

Patients with histologically confirmed SBCs who were treated between December 31, 2003, and January 31, 2019, were included in the present study. The patients’ clinical data and follow-up information were retrospectively reviewed. This study was approved by the ethics committee of Beijing Tiantan Hospital (KY2018-053–02). All patients signed informed consent forms for surgery treatment pre-operatively. Because of the retrospective nature of the present study and that the results will not affect the treatment strategy for patients, no additional informed consent was required to participate in the study.

### Radiological evaluation

**Fig. 1:**
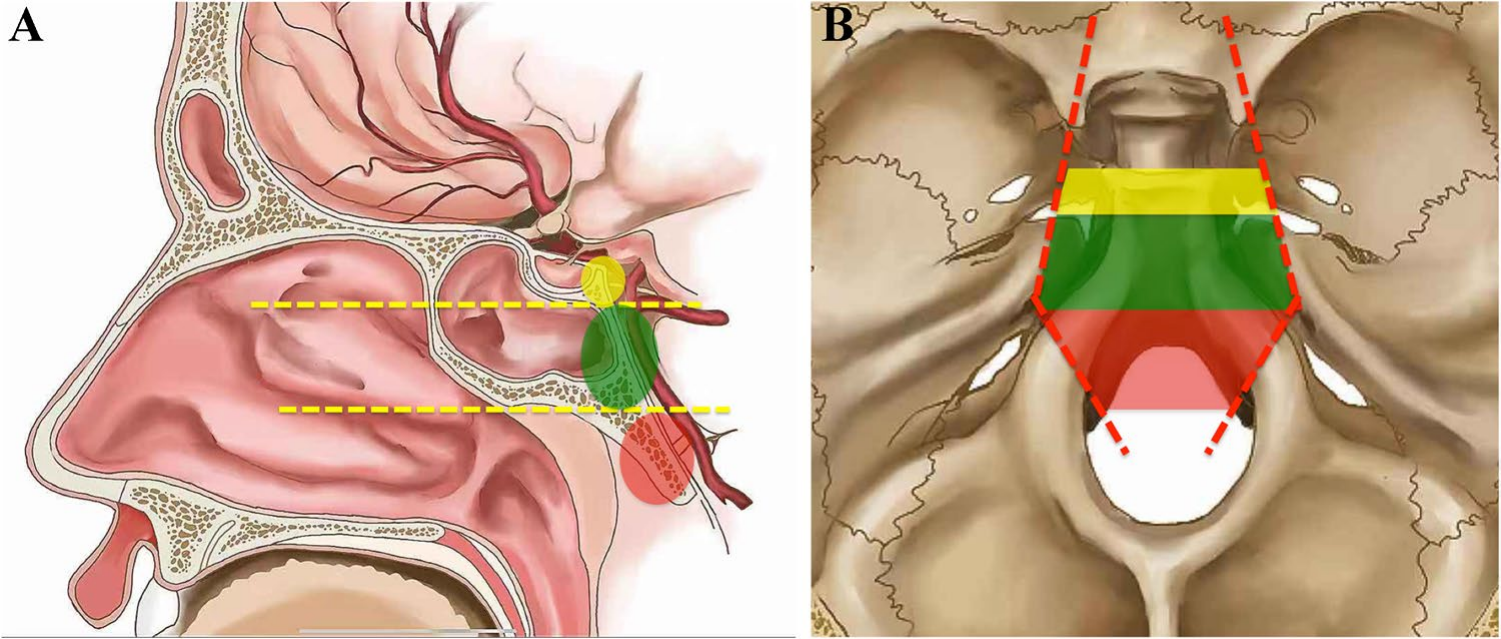
Anatomic classification of the clival region. **A** Sagittal view, the upper yellow horizontal line represents the sellar floor plane; the lower yellow horizontal line represents the sphenoid sinus floor plane. The yellow part represents the superior clivus; the green part represents the middle clivus; and the red part represents the inferior clivus. **B** Axial view, the red lines represent the bilateral lines connecting the lateral wall of the cavernous sinus, internal auditory canal, hypoglossal nerve hole, and occipital condyle. The region between the red line is the midline region of the skull base, and the region beyond the red line is the paramedian region

All patients underwent MRI and CT examinations pre-operatively. The MRI sequences mainly include T1-weighted images in the sagittal and axial planes, T2-weighted images in the axial plane, and postcontrast T1-weighted images in the sagittal, axial, and coronal planes. The sequence parameters were as follows: T1WI (axial), FOV 240 × 240, 24 slices, TE = 28.16 ms, TR = 2275 ms, and TI (inversion time) = 950 ms; T2WI, FOV 240 × 240, 24 slices, TE = 147 ms, TR = 8000 ms, and TI (inversion time) = 2000 ms; and DWI, FOV 240 × 240, 24 slices, TE = 63.2 ms, and TR = 2300 ms. Each patients’ pre- and post-operative images were evaluated by both radiologist on duty and our neuroendoscopic team. The tumor volume was calculated as volume = (a × b × c)/2, where a, b, and c represent the longest diameters in sagittal, coronal, and axial views, respectively. According to the sellar floor and sphenoid sinus floor plane in sagittal view, the clivus was divided into the superior, middle, and inferior zones, as previously described by our group (Fig. 1A) [12]. According to the bilateral boundaries traditionally established for EMA, the skull base was divided into midline and paramedian regions (Fig. 1B) [12]. When > ½ circumference of the internal carotid artery (ICA) was encased, cavernous sinus invasion was defined. Tumors that had invaded into the subdural space were defined as exhibiting dural penetration [36].

### Surgical methods

We used both EMAs (transnasal or transoral or combined) and MLOAs, which were chosen according to the tumor location [12]. When a tumor was located in the midline region or was slightly extended to the paramedian region, EMA was the first choice. When the tumor was mainly located in the paramedian region, we chose MLOA. Based on our belief that the dura is a natural barrier against intracranial invasion by chordomas, we did not cut off the inner layer of dura but thoroughly resected the tumor tissue that attached to the dura in patients with primary chordomas. When dural penetration existed [36], we followed the tumor passage and resected it. For patients with primary chordomas, a typical multi-layer reconstruction of clival defects was consisted of inlay collagen substitutes and autologous fat pad, onlay autologous and fascia lata, and vascularized nasoseptal flap. For patients with recurrent chordomas whose nasoseptal flap was not available, repairing the defects with absorbable collagen substitutes and autologous fascia lata was usually effective to prevent CSF leakage. In a few complicated cases, such as the patients have RT history and the defects were larger, we would deliberately preserve the most inner layer of chordoma in order to avoid high-flow CSF leak, which is a balance between the risk of repairing failure and the quality of life. When the tumor volume that invaded into subdural space was large and the dural defect after tumor removal was large and no vascularized flap was available, we will suture the autologous fat pad to normal dural edge using 5–0 prolene stitches besides the above-mentioned multi-layer reconstruction method. If refractory CSF happened, pedicle temporalis muscle flap was extremely useful. Post-operative lumbar drainage was generally placed for 5 days for patients with large defects. The resection rate was divided into four grades according to the postsurgical MRI and CT combined with the intraoperative impressions of surgeons [30]. CT images were scanned on the night of surgery day, and MR images were acquired within 2 days after operation except severe complications happen. Gross-total resection (GTR) indicated that the entire tumor was resected, the surrounding healthy tissue was obviously exposed, and no suspected tumor could be found on postsurgical images. No residual tumor was found during surgery, and > 90% tumoral resection on images was defined as near-total resection (NTR); > 70% tumoral resection on images was defined as subtotal resection (STR), and a lesser extent of resection was defined as partial resection (PR). For the sake of comparison, GTR and NTR were pooled and classified as marginal resection, and STR and PR were classified as intralesional resection [24, 25, 30].

### Follow-up

The first follow-up was performed in outpatient center after surgery. Thereafter, enhanced MRI was done every 6 months. RT was recommended to all patients. The date of the last follow-up, which was conducted by telephone, was November 9, 2019. Inquiries were made regarding RT modalities and dates. RT modalities include radiosurgery (Gamma Knife and Cyberknife), intensity-modulated photon radiotherapy (IMRT), and charged particle radiotherapy (CPRT, which includes both proton and carbon ion RT). Tumor recurrence was confirmed by radiological imaging. Progression-free survival (PFS) was calculated from the date of surgery to the date of radiographic recurrence. Chordoma-specific survival (CSS) was defined as the time between the date of surgery and the date of death caused by chordoma. If a patient was lost to follow-up or died of surgical complications or non-disease-specific reasons, censor data were applied.

### Statistical analyses

Statistical analyses were performed using SPSS 19 (IBM Corp, Armonk, New York) or R version 3.6.1 (R Foundation for Statistical Computing, Vienna, Austria). The difference in age distributions between groups was tested using the Mann–Whitney U test. PFS and CSS were estimated with the Kaplan–Meier method. To evaluate the influence of RT timing on CSS, patients were classified into three categories: RT before recurrence (pre-recurrence RT), RT after recurrence (late RT), and no RT. A univariate Cox proportional hazards model was performed to evaluate the marginal association between a potential prognostic factor and CSS/PFS. For factors with *P* value ≤ 0.05 in marginal analyses, we performed multivariate Cox proportional hazards regression to jointly estimate the effect of these factors. We used χ^2^ test to evaluate the association between factors and the resection rate; factors with *P* ≤ 0.05 were then jointly analyzed using logistic regression. Factors with *P* < 0.05 in joint analyses were considered statistically significant.

## Results

### Patients’ demographic and clinical characteristics

A total of 284 patients underwent 382 surgeries (Table 1), including 380 skull base surgeries (349 EMAs and 31 MLOAs) and two metastatic lesion resections. Common presenting symptoms included diplopia (50.7%), headache or neck pain (34.5%), and blurry vision (24.6%). The median duration from self-reported initial symptoms to diagnosis was 6.0 months (range 1.0–108.0 months). A total of 184 patients were newly diagnosed, and 100 patients had a treatment history. Marginal resection was achieved for 74.6% of patients, including 114 (40.1%) undergoing GTR and 98 (34.5%) undergoing NTR. Intralesional resection was achieved in 25.4% of tumors, including 64 (22.5%) with STR and eight (2.8%) with PR. A total of 111 patients underwent postsurgical RT, including 64 patients with pre-recurrence RT (mean time between surgery and RT, 3.5 months; range, 1–13 months) and 47 patients with late RT (mean time, 21.7 months; range, 4–79 months).

**Table 1:** Demographic and clinical characteristics of 284 patients with clivus chordomas

		No. (%)
Sex	Male	162 (57.0%)
	Female	122 (43.0%)
Tumor status	Primary	184 (64.8%)
	Recurrent	100 (35.2%)
Age, years ^a^	Median (range)	44.0 (3.0–77.0)
	Male	44.0 (5.0–76.0)
	Female	43.5 (3.0–77.0)
	Primary	44.0 (3.0–77.0)
	Recurrent	43.5 (3.5–70.0)
RT history	Yes	47 ^b^ (16.5%)
	No	237 (83.5%)
Previous RT modality	Radiosurgery	41 (87.2%)
	IMRT	2 (4.3%)
	CPRT	4 (8.5%)
Postsurgery RT	Yes (pre-recurrence RT)	64 (22.5%)
	Yes (late RT)	47 (16.5%)
	No	147 (51.8%)
	NA	26 (9.2%)
Postsurgery RT modality	Radiosurgery	40 (36.0%)
	IMRT	33 (29.7%)
	CPRT	31 (27.9%)
	NA	7 (6.3%)
Tumor volume median (range) (cm^3^)		22.3 (0.91–258.0)
Dural penetration	Yes	138 (48.6%)
	No	136 (47.9%)
	Unclear ^c^	10 (3.5%)
Histologic subtype	Conventional	216 (76.1%)
	Chondroid	63 (22.2%)
	Dedifferentiated ^d^	5 (1.8%)
Metastasis	Yes	9 (3.2%)
	No	242 (85.2%)
	NA	33 (11.6%)

^a^ No significant difference between sexes (*P* = 0.65) or between primary and recurrent tumors (*P* = 0.61). ^b^ Including 17 patients with radiotherapy as initial treatment, and 30 patients with recurrent tumor who have underwent surgeries and followed by RT treatment previously. ^c^ Unclear, difficult to judge by radiologic evaluation and surgical records. ^d^ Included four dedifferentiated chordomas and one sarcomatoid chordoma. *RT*, radiation therapy. *NA*, not available. *IMRT*, intensity-modulated photon radiotherapy. *CPRT*, charged particle radiotherapy

### Factors associated with CSS

**Fig. 2:**
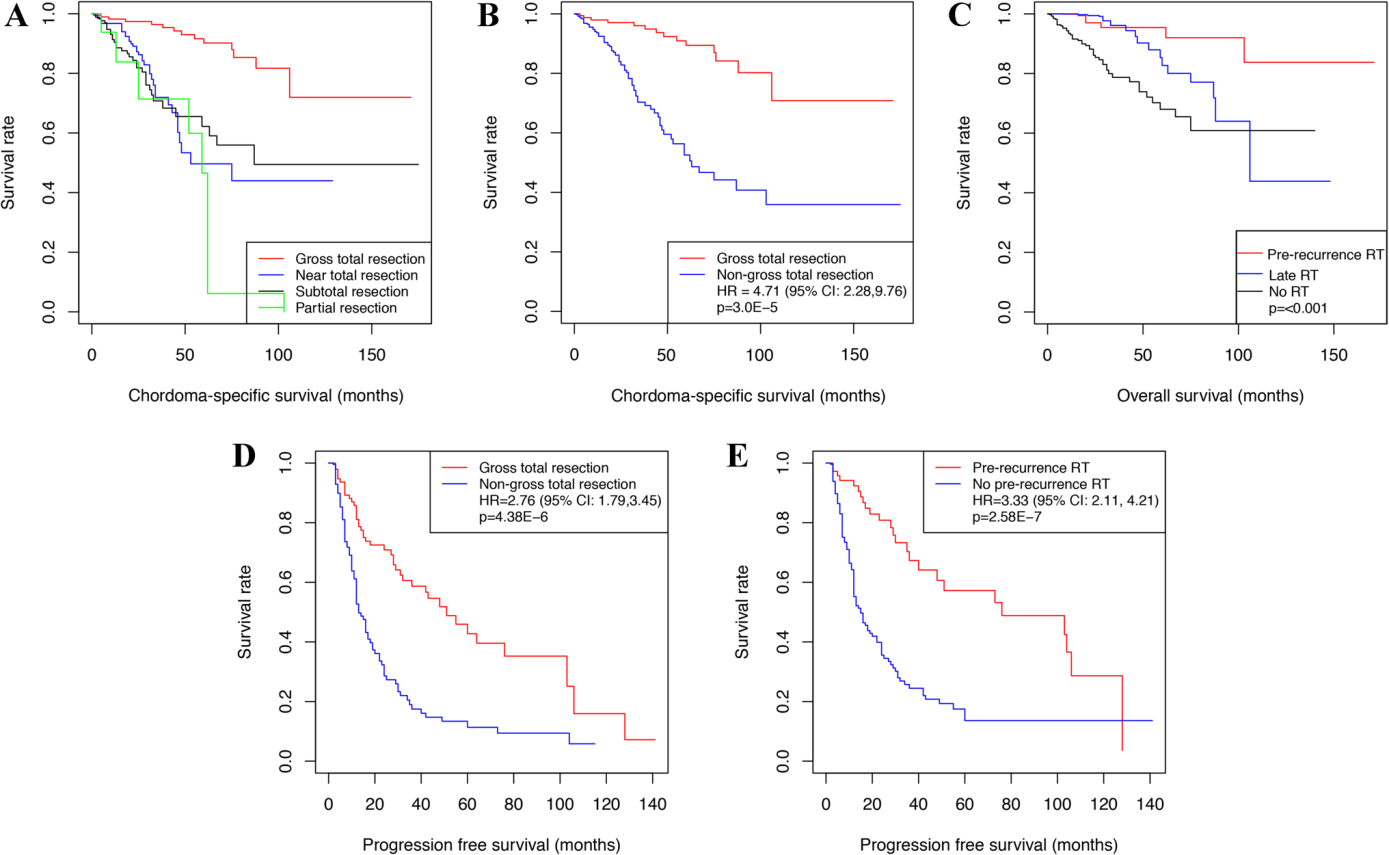
Risk factors for chordoma-specific survival (CSS) and progression-free survival (PFS) in all 284 patients. **A** The CSS was significantly longer in the gross-total resection (GTR) group than in the near-total resection (NTR) group, subtotal resection (STR) group, or partial resection (PR) group (*P* < .05). **B** When the NTR group, STR group, and PR group were classified into the non-gross-total resection group (non-GTR), the CSS was longer in the GTR group than in the non-GTR group (*P* < .05). **C** The group with pre-recurrence radiation therapy (RT) had significantly longer CSS than both the group without RT and the group with late RT (*P* < .05). **D** PFS was longer in the GTR group than in the non-GTR group (*P* < .05). **E** The patients who underwent RT before recurrence (pre-recurrence RT) had a longer PFS than the other patients (no pre-recurrence RT) (*P* < .05)

The average follow-up time was 43.9 months (median 32.5, range 2–175). At the last follow-up, 181 patients were alive. The 5-year CSS was 71.0% (95% CI = 62.8–80.2%). The patients with marginal resection had significantly longer survival times than those with intralesional resection (HR = 0.18, 95% CI = 0.089–0.360, *P* = 1.5 × 10^–6^). We performed a more detailed analysis and found that GTR significantly improved survival, while other groups (i.e., NTR, STR, and PR) did not show significant differences (Fig. 2A).

We found no significant difference in CSS between conventional and chondroid chordomas (HR = 1.57, 95% CI = 0.70–3.49, *P* = 0.273). For clarity, we combined conventional and chondroid chordomas into one group and classified the resection rate into GTR and non-GTR. The results based on univariate analysis are reported in Supplementary Table 1. The 5-year CSS was higher in the GTR group [90.3% (95% CI = 83.6–97.6%)] than in the non-GTR group [49.8% (95% CI = 38.8–63.8%)] (Fig. 2B). The group with pre-recurrence RT had significantly longer CSS than both the group without RT and the group with late RT (Fig. 2C). Based on the multivariate Cox proportional hazards model, we found that female sex (HR = 1.65, 95% CI = 1.02–2.68, *P* = 0.043), dedifferentiated subtype (HR = 8.21, 95% CI = 2.27–29.67, *P* = 0.001), history of RT (HR = 2.04, 95% CI = 1.06–3.93, *P* = 0.034), non-GTR (HR = 4.71, 95% CI = 2.28–9.76, *P* < 0.001), tumor location of lower 2/3 clivus (HR = 2.32, 95% CI = 1.15–4.67, *P* = 0.018), cavernous invasion (HR = 2.16, 95% CI = 1.16–4.05, *P* = 0.016), and no postsurgical RT (HR = 4.87, 95% CI = 2.03–11.65, *P* < 0.001) and metastasis (HR = 5.39, 95% CI = 1.96–14.84, *P* = 0.001) were independent risk factors for CSS (Table 2).

**Table 2:** Factors associated with chordoma-specific survival in patients with skull base chordomas. Analysis was based on a multivariate Cox proportional hazards model

Variable		Hazard ratio	95% CI	*P* value
Sex	Female vs. male	1.65	1.02–2.68	.**043**
Age ^a^	Group 2 vs. group 1	1.77	0.79–3.95	.162
	Group 3 vs. group 1	1.67	0.62–4.53	.314
Histopathologic subtype	Dedifferentiated vs. conventional/chondroid	8.21	2.27–29.67	.**001**
History of surgery	Yes vs. no	1.45	0.75–2.79	.273
History of RT	Yes vs. no	2.04	1.06–3.93	.**034**
Resection rate	Non-GTR vs. GTR	4.71	2.28–9.76	** < **.**001**
Tumor location (Sagittal view)	Lower 2/3 vs. upper 2/3	2.32	1.15–4.67	.**018**
	Total clivus vs. upper 2/3	1.17	0.64–2.14	.603
	Others vs. upper 2/3	0.38	0.08–1.72	.207
Tumor location (Axial view) ^b^	Median extension to the paramedian region vs. the midline	0.70	0.40–1.21	.197
Cavernous sinus invasion	Yes vs. no	2.16	1.16–4.05	**.016**
Dural penetration	Yes vs. no	1.35	0.79–2.32	.274
Tumor volume	≤ 40 vs. > 40	0.67	0.38–1.21	.187
Postsurgical RT	Late RT vs. pre-recurrence RT	2.02	0.76–5.36	.160
	No RT vs. pre-recurrence RT	4.87	2.03–11.65	< .**001**
Metastasis	Yes vs. no	5.39	1.96–14.84	.**001**

^a^ Age was classified into three groups, e.g., group 1 represented ≤ 20 years of age, group 2 represented between 20 and 60 years of age, and group C represented ≥ 60 years of age. ^b^ One patient whose tumor was located in the paramedian region was included in the group with median extension to the paramedian region

The bolded entries are significant *p*-values

### Risk factors for recurrence

During the follow-up, 155 patients (54.6%) had tumor recurrence or progression of residual tumor, and 90 patients (31.7%) had no tumor recurrence. The accurate PFS of 39 patients (13.7%) was not available. The mean PFS was estimated to be 46.7 months (range, 2–141 months). The results of the univariable analysis for PFS are shown in Supplementary Table 1. Based on the multivariate Cox proportional hazards model, we found that non-GTR (HR = 2.76, 95% CI = 1.79–3.45, *P* < 0.001) (Fig. 2D), no pre-recurrence RT (HR = 3.33, 95% CI = 2.11–4.21, *P* < 0.001) (Fig. 2E), history of RT (HR = 1.80, 95% CI = 1.05–2.37, *P* = 0.033), and history of surgery (HR = 1.59, 95% CI = 1.04–1.97, *P* = 0.034) were associated with shorter PFS (Table 3).

**Table 3:** Factors associated with progression-free survival in patients with skull base chordomas. Analysis was based on a multivariate Cox proportional hazards model

Variable		Hazard ratio	95% CI	*P*
Sex	Female vs. male	0.99	0.69–1.20	.971
Age ^a^	Group 2 vs. group 1	1.53	0.93–1.98	.097
	Group 3 vs. group 1	1.44	0.75–2.01	.274
Histopathologic subtype	Dedifferentiated vs. conventional/chondroid	2.40	0.65–4.65	.188
History of surgery	Yes vs. no	1.59	1.04–1.97	**.034**
History of RT	Yes vs. no	1.80	1.05–2.37	**.033**
Resection rate	Non-GTR vs. GTR	2.76	1.79–3.45	** < .001**
Tumor location (sagittal view)	Lower 2/3 vs. upper 2/3	1.05	0.65–1.35	.836
	Total clivus vs. upper 2/3	0.93	0.59–1.18	.760
	Others vs. upper 2/3	0.53	0.20–0.86	.193
Tumor location (axial view) ^b^	Paramedian vs. midline	1.23	0.83–1.51	.295
Cavernous sinus invasion	Yes vs. no	1.13	0.73–1.41	.588
Dural penetration	Yes vs. no	0.85	0.59–1.02	.378
Tumor volume	> 40 vs. ≤ 40 cm^3^	1.03	0.67–1.28	.889
Postsurgical RT	No RT vs. RT	3.33	2.11–4.21	** < .001**

^a^ Age was classified into three groups, e.g., group 1 represented ≤ 20 years of age, group 2 represented between 20 and 60 years of age, and group 3 represented ≥ 60 years of age. ^b^ One patient whose tumor was located in the paramedian region was included in the group with median extension to the paramedian region

The bolded entries are significant *p*-values

### Factors associated with long-term outcomes in primary SBCs

**Fig. 3:**
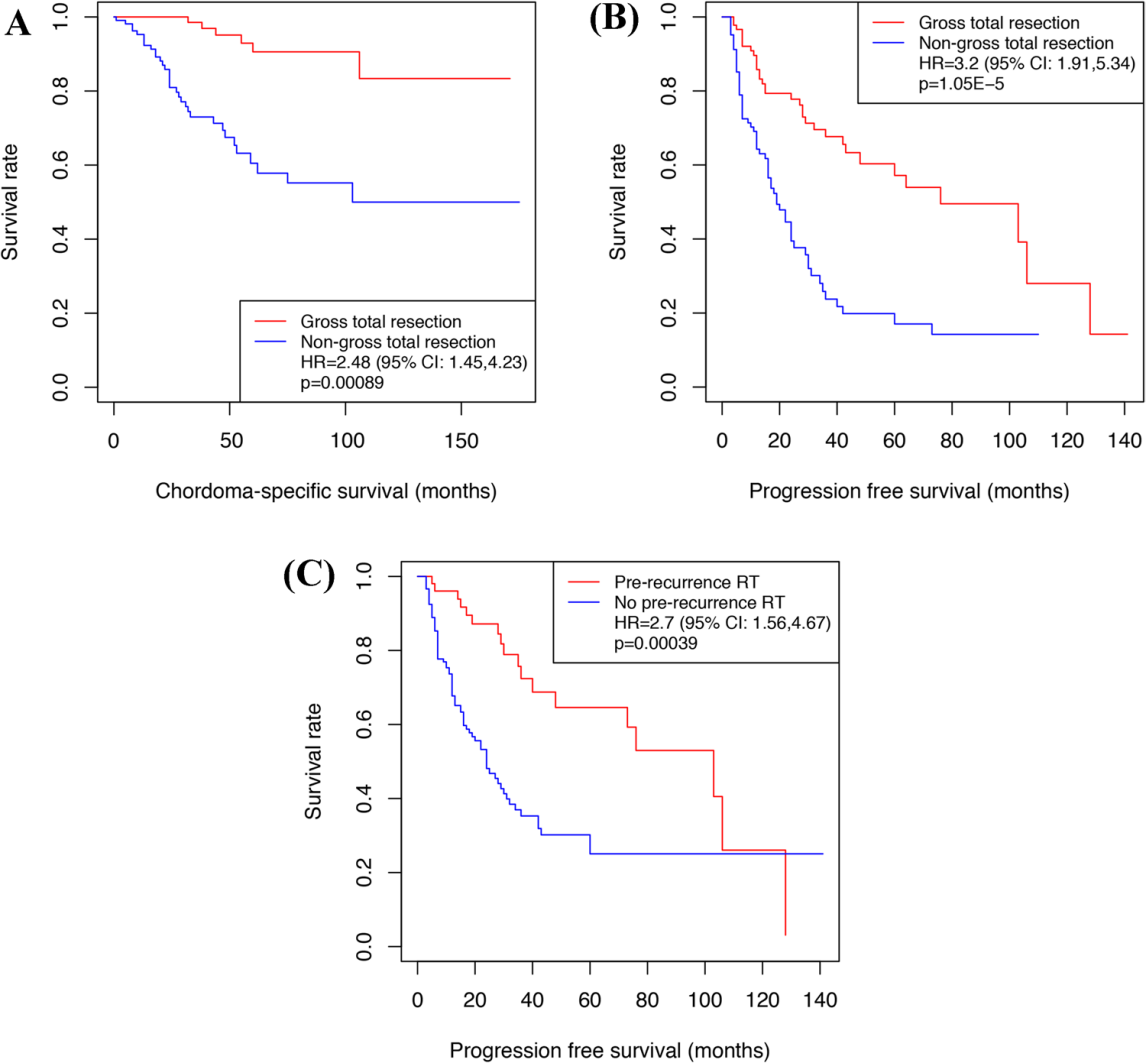
Risk factors for chordoma-specific survival (CSS) and tumor recurrence in 184 primary chordoma patients. **A** Non-gross-total resection (non-GTR) was statistically associated with poorer CSS (*P* < .001).** B** Non-GTR (*P* < .001) and **C** no pre-recurrence RT (*P* < .001) significantly increased the risk of recurrence

To further clarify the effect of risk factors for CSS and PFS, we restricted analyses to 184 patients with primary tumors. The results showed that non-GTR (HR = 2.48, 95% CI = 1.45–4.23, *P* < 0.001) (Fig. 3A), tumor location in the lower 2/3 of the clivus (HR = 2.54, 95% CI = 1.32–4.87, *P* = 0.005), cavernous invasion (HR = 1.96, 95% CI = 1.03–3.70, *P* = 0.041), late RT (HR = 2.77, 95% CI = 1.43–5.35, *P* = 0.002), and no RT (HR = 1.98, 95% CI = 1.09–3.60, *P* = 0.026) were statistically associated with poor CSS. Moreover, non-GTR (HR = 3.20, 95% CI = 1.91–5.37, *P* < 0.001) (Fig. 3B) and no pre-recurrence RT (HR = 2.70, 95% CI = 1.56–4.67, *P* < 0.001) (Fig. 3C) significantly increased the risk of recurrence.

### Factors associated with the failure of GTR

Given the importance of GTR, we performed a multivariate logistic regression analysis to identify factors that are associated with the failure of GTR. We found that younger age (OR = 0.28, 95% CI = 0.09–0.87, *P* = 0.028), history of surgery (OR = 3.45, 95% CI = 1.59–7.49, *P* = 0.002), tumor location (lower 2/3 of the clivus, OR = 8.73, 95% CI = 3.12–24.45, *P* < 0.001; total clivus, OR = 3.07, 95% CI = 1.36–6.94, *P* = 0.007; invasion into the paramedian region, OR = 2.88, 95% CI = 1.42–5.82, *P* = 0.003), and large tumor volume (> 40 cm^3^) (OR = 3.07, 95% CI = 1.22–7.76, *P* = 0.018) were associated with the failure of GTR (Table 4) (see also Supplementary Table 2 for univariate logistic regression analyses). The GTR rate showed no significant difference between different approaches (EMA 40.3% and MLOA 37.5%, *P* = 0.82) (Supplementary Table 2).

**Table 4:** Risk factors associated with failure of gross-total resection. Analysis based on multivariable logistic regression

Variable		OR	95% CI	*P*
Sex	Female vs. male	1.49	0.78–2.85	.223
Age ^a^	Group 2 vs. group 1	0.28	0.09–0.87	**.028**
	Group 3 vs. group 1	0.27	0.07–1.02	.054
Histopathologic subtype	Dedifferentiated vs. conventional/chondroid	1.35	0.09–20.19	.826
History of surgery	Yes vs. no	3.45	1.59–7.49	**.002**
History of RT	Yes vs. no	0.80	0.27–2.34	.686
Tumor location (sagittal view)	Lower 2/3 vs. upper 2/3	8.73	3.12–24.45	** < .001**
	Total clivus vs. upper 2/3	3.07	1.36–6.94	**.007**
	Others vs. upper 2/3	1.66	0.30–9.16	.561
Tumor location (axial view) ^a^	Paramedian vs. midline	2.88	1.42–5.82	**.003**
Cavernous sinus invasion	Yes vs. no	1.72	0.81–3.67	.158
Dural penetration	Yes vs. no	1.73	0.90–3.32	.101
Tumor volume	> 40 vs. ≤ 40 cm^3^	3.07	1.22–7.76	**.018**

^a^ Age was classified into three groups, e.g., group 1 represented ≤ 20 years of age, group 2 represented between 20 and 60 years of age, and group C represented ≥ 60 years of age. ^b^ One patient whose tumor was located in the paramedian region was included in the group with median extension to the paramedian region

The bolded entries are significant *p*-values

### Surgical complications

Complications were comprised of the following: cranial nerve injury (7.9%), cerebrospinal fluid (CSF) leakage (3.9%), intracranial infection (3.2%), hypopituitarism (2.1%), severe pneumonia followed by lower cranial injury (1.6%), postsurgical hematoma (0.5%), and hydrocephalus (0.5%). ICA injury occurred in seven patients (1.8%), and two of them were treated with endovascular treatment [39]. Six patients (1.6%) died of complications. The complications were also compared between EMA and MLOA (Supplementary Table 3).

To be mentioned, two patients have CSF leakage after RT. One of them was treated by lumbar drainage, and another one was treated by transnasal endoscopic repair surgery.

### Effects of RT on long-term outcomes when GTR was achieved

A total of 89 patients with primary SBCs achieved GTR. PFS was significantly longer in the group with pre-recurrence RT than in the others (HR = 3.41, 95% CI = 1.39–8.35, *P* = 0.007) after adjusting for age, sex, and tumor volume. CSS was not evaluated due to the limited number of death events.

Similarly, we analyzed the effect of RT in 95 patients with primary SBCs who did not achieve GTR. Compared with no pre-recurrence RT, pre-recurrence RT also showed significant positive effect on PFS (HR = 2.50, 95% CI = 1.30–4.84, *P* = 0.006).

### Radiotherapy modalities and outcomes

To study the relationship between RT modalities and outcomes, we analyzed 45 patients with primary SBCs who were treated with either CPRT or other RT modalities. However, because of the limited statistical power due to the short follow-up and limited number of cases in the group with CPRT, we did not observe significant differences in CSS or PFS between the two groups (Supplementary Table 4).

## Discussion

### Choice of surgical approach

In present series, both EMA and MLOA were used to resect SBCs. We did not find a significant difference in the GTR rate between EMA and MLOA, which was consistent with the previous findings of our group and others [30, 34, 37]. To be mentioned, most of our cases were resected through EMAs, which is mainly determined by tumor’s origin sites and extending directions [10, 23, 26]. EMA has advantage in reducing injury of cranial nerve compared with MLOA, because the tumor can be safely resected without crossing the nerves in most circumstances [17]. However, the most serious and urgent complication is ICA injury. A strategy for expanding exposure for ICA must be planed carefully according to the tumor extending direction pre-operatively; meanwhile, an endovascular treatment should be available just in case for this rare complication [39]. Although seven cases (2.0%) experienced ICA injury in present series, they were recovered well except one patient with recurrent chordoma died of ICA occlusion in our early stage. In summary, we regard that EEA is the first choice for SBCs, and it is also safe when the surgeons have enough experience [7]. A neurosurgeon who majored in the treatment of SBCs should master both EMA and MLOA [1].

### Resection rate and outcome

The resection rate strongly influences CSS and PFS [8, 37, 38]. The maximum resection was traditionally recommended with the goal of marginal resection [24, 25, 30]. The recurrence rate ranges from 16 to 45% at 10 years after marginal resection, and most recurrences occur within 2 or 3 years [14, 23, 34]. After a more detailed analysis, we found that the advantage of marginal resection was mainly contributed by GTR and that no obvious differences were found among NTR, STR, and PR. Therefore, GTR should be the goal if possible [23]. This result also indicates that the standard for assessing the resection rate should be postsurgical images rather than the intraoperative impression of the surgeon [6].

### Other risk factors for outcome

Dedifferentiated subtype, history of RT, lack of postsurgical RT, and metastasis increased the risk of both shorter survival and recurrence, which has been shown by previous studies [5, 8, 10, 17, 34]. However, it is controversial whether different survival rates exist between conventional and chondroid types [7, 29]. We found no significant difference, which is consistent with several studies [2, 32]. Several previous studies showed that dural penetration [7, 36] and age [5, 29] were associated with long-term outcomes, and we demonstrated that they were not independent risk factors after multivariate analysis [23, 38].

### Risk factors reducing the chances of GTR

We found that younger age (≤ 20 years) increased the risk of non-GTR. This may be explained by our previous finding that most children with SBCs had these tumors in the lower clivus and craniocervical junction area [3]. Tumors in the lower clivus have the lowest GTR rate [3, 6, 14, 17]. Not surprisingly, history of surgery, large tumor volume, and tumor location are independent risk factors for GTR, as supported by previous studies [7, 14, 17, 26, 30].

### Surgical complications and treatments

CSF leakage and cranial nerve injury were the main complications in prior studies [6, 14, 17]. Benefiting mainly from the application of vascularized nasoseptal flaps [7, 17], CSF leakage was low in our group (3.9%). We emphasize the importance of cleaning tumors that are attached to the dura while maintaining the integrity of the dura, which effectively prevent CSF leakage. Radical resection of all involved dura might account for high rates of CSF leakage (14%) [6, 9]. We deem the dura to be a natural barrier against tumor growth intracranially, which has been supported indirectly by two autopsy studies [21, 28]. Both studies found that the dura was intact even in advanced stages of SBCs.

Chordomas with cavernous sinus invasion have a lower GTR rate (23.0% vs 47.7%), although it is not an independent risk factor. The major difficulty for achieving GTR in this setting was fear of ICA injury, which is another serious complication [17]. Endovascular treatment is an effective remedial treatment when the ICA is injured [39]. Therefore, we support the notion that EMA is safe and has few complications once adequate experience is gained [26].

### Timing of RT and outcome

Adjuvant RT is a widely accepted treatment for residual chordomas [19, 40], although no strong evidence supports it [31]. We found that PFS and CSS were significantly longer in patients with pre-recurrence RT than in other patients, which is similar to prior studies [14, 34]. In addition, the PFS in the late RT group was shortest in the present series (data not shown); however, the CSS was still longer than that of the patients who had no RT. Similarly, Olabisi Sanusi et al. found that a second dose of RT at any point either as sole treatment or as adjuvant treatment for recurrence showed a statistically significant effect on PFS (P = 0.009) [22]. Taken together, these findings indicate that adjuvant RT is an independent factor for better outcomes [7, 34], and the importance of pre-recurrence RT is worth emphasizing. Once recurrence occurs, postsurgical RT is also recommended to prolong CSS when the patient has no RT history [25].

### RT after GTR

As expected, our finding of pre-recurrence RT in patients who failed GTR strengthens the positive role of RT in postponing recurrence [7, 22]. However, it remains debatable whether to undertake pre-recurrence RT after GTR [8, 19, 23]. Lately, Yagiz Yolcu et al.’s found that compared with GTR alone, GTR plus RT did not offer any significant survival benefit for patients with sacral or spinal chordomas, at the price of higher complications rate [33]. However, our result is consistent with a recent finding that pre-recurrence RT in GTR group will prolong PFS [22]. Therefore, pre-recurrence RT is recommended to prevent recurrence even after GTR for patients with SBCs [19]. 

### Radiation modalities and outcome

In recent years, increasing studies found that CPRT has advantages over other modalities in the chordoma treatment. Satoshi Takahashi et al. [27] found that the PFS of carbon ion treated group was longer than that of the other groups treated with other radiation modalities or untreated; however, a recent meta-analysis did not find significant differences between radiosurgery, proton RT, and carbon ion RT at 3 and 5 years survival [40]. Li H et al. also found that IMRT serves as an effective alternative to CPRT based on their retrospective analysis of 46 cases. Selection bias was likely present in Li’s study and might be conducive to a result that contradicts previous findings; for example, economic status (CPRT is much more expensive than other RT modalities) and education status (which will affect whether the patients would follow the clinical recommendation) influence the accessibility of CPRT. For now, we cannot compare the effectiveness of CPRT and other radiation modalities due to short follow-up and limited number of cases with CPRT. Thus, we cannot rule out the possibility that there is no benefit of CPRT over other types of radiation, which might be associated with the improved RT techniques of radiosurgery or IMRT [15, 16]. Although we recommend that patients with chordomas seek help from radiologists who administer particle radiation therapy, we firmly believe that a randomized trial is warranted even in its rarity [14].

### Limitations

As a retrospective study, our study has multiple limitations, including the lack of well-designed timing of RT and random choice of radiation modalities. In addition, we cannot assess whether pre-recurrence RT improves CCS in patients who have achieved GTR (n = 114) due to the limited number of death events (n = 14) in the current study. We will continue following up in order to evaluate the effect of pre-recurrence RT. Moreover, tumor biological characteristics were not analyzed; these characteristics might be underlying factors for GTR [8]. Recently, we found that genomic alterations were associated with recurrence and CSS [4]. Therefore, combining tumor biology with clinical characteristics will produce more robust conclusions in the future.

## Conclusions

To the best of our knowledge, this is the largest series of SBCs surgically treated by single surgical team, and we found that surgery should be the initial treatment for primary SBCs. GTR is the surgical goal and should be applied in both primary and recurrent SBCs [38]. History of surgery, larger tumor volume, and tumor location (lower clivus, extension from the midline to the paramedian region) are independent risk factors for GTR. Pre-recurrence RT will postpone recurrence, even after GTR is achieved.

## Supplementary Information

Below is the link to the electronic supplementary material.

ESM 1 (85.8 KB)

## Data Availability

Not applicable.
