# Supplementary material for: Mid-term follow-up surgical results in 284 cases of clival chordomas: the risk factors for outcome and tumor recurrence
Source: Neurosurg Rev. 2021 Oct 8;45(2):1451–62. doi: 10.1007/s10143-021-01576-4 (PMC8976789; doi:10.1007/s10143-021-01576-4)
Supplement: Supplementary file 1 — (85.8 KB) [file 10143_2021_1576_MOESM1_ESM.docx]

Supplementary Table 1. The results of chordoma-specific survival (CSS) and progression-free survival (PFS) with possible risk factors by univariable analysis

| **Risk Factors** | **No.** | **CSS (months) mean, 95% CI** | ***P*** | **No.** | **PFS (months) mean, 95% CI** | ***P*** |
| --- | --- | --- | --- | --- | --- | --- |
| **Sex** |  |  |  |  |  |  |
| **Male** | 162 | 111.4 (96.3, 126.5) | **.006** | 146 | 45.9 (36.2 - 55.7) | .515 |
| **Female** | 122 | 92.8 (76.7, 108.9) |  | 99 | 43.0 (33.1 - 52.9) |  |
| **Age** |  |  |  |  |  |  |
| **≤ 20** | 34 | 92.4 (73.1, 111.7) | .681 | 34 | 48.8 (30.5 - 67.1) | .772 |
| **20-60** | 209 | 103.4 (90.6, 116.2) |  | 177 | 47.4 (38.2 - 56.6) |  |
| **≥60** | 41 | 88.8 (68.0, 109.6) |  | 34 | 36.1 (23.8 -48.4) |  |
| **History of surgery** |  |  |  |  |  |  |
| **Yes** | 91 | 55.4 (42.4, 68.3) | **1.2102E-9** | 71 | 22.2 (39.1 - 54.3) | **5.0034E-9** |
| **No** | 193 | 124.4 (111.7, 137.1) |  | 174 | 56.0 (46.5 - 65.5) |  |
| **History of radiotherapy** |  |  |  |  |  |  |
| **Yes** | 47 | 37.1 (26.9, 47.3) | **2.0334E-14** | 34 | 18.8 (8.1 - 28.4) | **.000002** |
| **No** | 237 | 120.6 (108.6, 132.6) |  | 211 | 50.9 (42.5 - 59.4) |  |
| **Dural penetration** |  |  |  |  |  |  |
| **Yes** | 138 | 71.8 (59.8, 83.8) | **.0002** | 115 | 42.5 (31.5 - 53.5) | .177 |
| **No** | 136 | 127.7 (112.9, 142.6) |  | 121 | 50.5 (40.0 - 61.0) |  |
| **Not clear ^a^** | 10 | 67.1 (40.8, 93.5) |  | 9 | 35.1 (8.8 - 61.4) |  |
| **Tumor volume** |  |  |  |  |  |  |
| **≤ 40 cm3** | 212 | 111.8 (99.0, 124.5) | **.006** | 182 | 53.1 (43.7 - 62.4) | **.0002** |
| **＞ 40 cm3** | 72 | 64.2 (50.9, 77.5) |  | 63 | 27.5 (18.2 - 36.9) |  |
| **Tumor locations (Sagittal view)** |  |  |  |  |  |  |
| **Upper2/3 ^b^** | 154 | 120.4 (105.5, 135.3) | **.001** | 134 | 55.7 (44.3 - 67.1) | **.014** |
| **Lower2/3** | 54 | 62.9 (47.9, 77.9) |  | 49 | 30.1 (18.6 - 41.5) |  |
| **Total clivus** | 67 | 64.3 (52.2, 76.4) |  | 54 | 36.4 (25.3 - 47.5) |  |
| **Others** | 9 | 95.1 (63.8, 126.4) |  | 8 | 50.6 (39.1 - 54.3) |  |
| **Tumor locations (Axial view)** |  |  |  |  |  |  |
| **Midline** | 154 | 119.2 (103.8, 134.7) | **.006** | 136 | 53.6 (42.5 - 64.8) | **.001** |
| **Midline extend to paramedian** | 129 | 76.6 (64.8, 88.4) |  | 108 | 32.8 (25.2 - 40.5_ |  |
| **Paramedian** | 1 | 110 (Alive) |  | 1 | 110 (No recurrence) |  |
| **Approach** |  |  |  |  |  |  |
| **Endoscopic midline** | 268 | 104.4 (92.6, 116.1) | .487 | 230 | 46.6 (38.7 - 54.5) | .937 |
| **Microscopic lateral open** | 16 | 103.6 (75.7, 131.4) |  | 15 | 37.9 (18.1 - 57.7) |  |
| **Cavernous sinus invasion** |  |  |  |  |  |  |
| **Yes** | 87 | 65.2 (50.8, 79.5) | **.000003** | 176 | 48.0 (39.1 - 56.9) | .086 |
| **No** | 197 | 118.8 (105.3, 132.2) |  | 69 | 39.0 (27.8 - 50.2) |  |
| **Resection Rate** |  |  |  |  |  |  |
| **GTR** | 114 | 140.5 (125.8, 155.2) | **1.5057E-10** | 100 | 71.9 (58.7 - 85.1) | **1.5622E-11** |
| **Non-GTR** | 170 | 78.6 (64.6, 92.6) |  | 145 | 27.2 (20.8 - 33.6) |  |
| **Post-Surgery Radiotherapy** | 258 |  |  |  |  |  |
| **No** | 147 | 82.6 (70.2, 94.9) | **.0002** | 172 | 38.8 (29.8 - 47.8) | **8.2206E-8** |
| **Yes (late RT)** | 47 | 96.3 (78.3, 114.2) |  |  |  |  |
| **Yes (pre-recurrence RT)** | 64 | 145.8 (127.9, 163.8) |  | 63 | 78.2 (64.0 - 92.5) |  |
| **Pathology subtype** |  |  |  |  |  |  |
| **Conventional** | 216 | 101.5 (89.0, 114.0) | **2.6938E-11** | 183 | 43.2 (35.2 - 51.3) | **.003** |
| **Chondroid** | 63 | 108.6 (92.7, 124.5) |  | 59 | 57.7 (41.1 - 74.2) |  |
| **Dedifferentiated ^C^** | 5 | 11.4 (2.36, 20.4) |  | 3 | 6.67 (4.31 - 9.02) |  |
| **Metastasis** |  |  |  |  |  |  |
| **Yes** | 9 | 51.3 (25.6, 77.1) | **.022** |  |  |  |
| **No** | 242 | 114.7 (103.0, 126.4) |  |  |  |  |
| **NA** | 33 | 81.2（59.4, 103.0） |  |  |  |  |

^a^ Not clear, difficult to judge by radiologic evaluation and surgical records. ^b^ Included the tumors that located simply in middle clivus; ^C^ Included four dedifferentiated chordomas and one sarcomatoid chordoma; NA, not available; RT, radiation therapy

Supplementary Table 2. Risk factors for failure of gross-total resection that analyzed with univariate logistic regression analyses

| **Parameter** | **No. of Patients (%)** | | | **Statistic Value** | **P** |
| --- | --- | --- | --- | --- | --- |
|  | Total | GTR | Non-GTR |  |  |
| **No. of total patients** | 284 | 114 (40.1%) | 170 (59.9%) |  |  |
| **Sex** |  |  |  |  |  |
| Male | 162 | 71 (43.8%) | 91 (56.2%) | Chi-Square 2.13 | .18 |
| Female | 122 | 43 (35.2%) | 79 (64.8%) |  |  |
| **Age** |  |  |  |  |  |
| ≤ 20 | 34 | 7 （20.6%） | 27 （79.4%） | Chi-Square 6.85 | **.03** |
| 20-60 | 209 | 87 （41.6%） | 122 （58.4%） |  |  |
| ≥60 | 41 | 20 （48.8%） | 21 （51.2%） |  |  |
| **History of surgery** |  |  |  |  |  |
| Yes | 91 | 20 (22.0%) | 71 (78.0%) | Chi-Square 18.39 | **＜.001** |
| No | 193 | 94 (48.7%） | 99 (51.3%) |  |  |
| **History of radiotherapy** |  |  |  |  |  |
| Yes | 47 | 10 (21.3%) | 37 (78.7%) | Chi-Square 8.34 | **.005** |
| No | 237 | 104 (43.9%) | 133 (56.1%) |  |  |
| **Dural penetration** |  |  |  |  |  |
| Yes | 138 | 35 (25.4%) | 103 (74.6%) | Chi-Square 25.29 | **＜ .001** |
| No | 136 | 75 (55.1%) | 61 (44.9%) |  |  |
| Not clear ^a^ | 10 | 4 (40.0%) | 6 (60.0%) |  |  |
| **Tumor volume** |  |  |  |  |  |
| ≤ 40 cm3 | 212 | 106 (50.0%) | 106 (50.0%) | Chi-Square 33.83 | **＜.001** |
| ＞ 40 cm3 | 72 | 8 (11.1%) | 64 (88.9%) |  |  |
| **Tumor locations (Sagittal view)** |  |  |  |  |  |
| Upper 2/3 ^b^ | 154 | 90 (58.4%) | 64 (41.6%) | Chi-Square 50.62 | **＜ .001** |
| Lower 2/3 | 54 | 6 (11.1%) | 48 (88.9%) |  |  |
| Total clivus | 67 | 14 (20.9%) | 53 (79.1%) |  |  |
| Others | 9 | 4 (44.4%) | 5 (55.6%) |  |  |
| **Tumor locations (Axial view)** |  |  |  |  |  |
| Midline | 154 | 91 (59.1%) | 63 (40.9%) | Chi-Square 50.23 | **＜ .001** |
| Midline extend to paramedian | 129 | 23 (17.8%) | 106 (82.2%) |  |  |
| Paramedian | 1 | 0 (0.0%) | 1 (100%) |  |  |
| **Approach** |  |  |  |  |  |
| Endoscopic midline | 268 | 108 (40.3%) | 160 (59.7%) | Chi-Square 0.05 | .82 |
| Microscopic lateral open | 16 | 6 (37.5%) | 10 (62.5%) |  |  |
| **Cavernous sinus invasion** |  |  |  |  |  |
| Yes | 87 | 20 (23.0%) | 67 (77.0%) | Chi-Square 15.36 | **＜ .001** |
| No | 197 | 94 (47.7%) | 103 (52.3%) |  |  |

^a^ Not clear, difficult to judge by radiologic evaluation and surgical records; ^b^ Included the tumors that located simply in middle clivus.

Supplementary Table 3. The comparison of complications between endoscopic midline approach (EMA) and microscopic lateral open approach (MLOA)

| Complications | Number of Cases, n (%) | | | *P* (EMA vs MLOA) |
| --- | --- | --- | --- | --- |
|  | EMA | MLOA | Total |  |
| CSF leakage | 14 (4.0%) | 1 (3.2%) | 15 (3.9%) | 1.000 |
| Hydrocephalus | 2 (0.6%) | 0 | 2 (0.5%) | 1.000 |
| Cranial nerves injury | 25 (7.2%) | 5 (16.1%) | 30 (7.9%) | 0.085 |
| III | 4 (1.1%) | 0 |  |  |
| IV | 0 | 1 (3.2%) |  |  |
| VI | 12 (3.4%) | 1 (3.2%) |  |  |
| VII | 1 (0.3%) | 1 (3.2%) |  |  |
| IX-XI | 8 (2.3%) | 2 (6.5%) |  |  |
| Intracranial infection | 8 (2.3%) | 4 (12.9%) | 12 (3.2%) | 0.011 |
| Hypopituitarism | 7 (2.0%) | 1 (3.2%) | 8 (2.1%) | 0.497 |
| Pneumonia | 4 (1.1%) | 2 (6.5%) | 6 (1.6%) | 0.079 |
| Hematoma | 1 (0.3%) | 1 (3.2%) | 2 (0.5%) | 0.157 |
| ICA injury | 7 (2.0%) | 0 | 7 (1.8%) | 1.000 |

Supplementary Table 4. The comparation of long-term outcomes between charged particle radiotherapy (CPRT) and other modalities with Kaplan-Meier analysis.

|  | **CPRT** | **Other modalities** | **Statistic Value** | ***P*** |
| --- | --- | --- | --- | --- |
| **No.** | 10 | 35 |  |  |
| **Follow-up (months) median, (range)** | 29.5 (9.0–42.0) | 51.0 (8.0-171.0) | 79 | .008 |
| **Chordoma Specific Survival** | No death | 157.3 (142.6-172.1) | .75 | .388 |
| **PFS (months)**  **mean, (95%CI)** | 29 ^a^ | 83.5 (66.0-101.0) | .48 | .488 |

^a^ n=1
